# Patients-people-place: developing a framework for researching organizational culture during health service redesign and change

**DOI:** 10.1186/s13012-014-0106-z

**Published:** 2014-08-20

**Authors:** Nicola K Gale, Jonathan Shapiro, Hugh S T McLeod, Sabi Redwood, Alistair Hewison

**Affiliations:** University of Birmingham, Health Services Management Centre, University of Birmingham, Edgbaston, Birmingham, B15 2RT UK; Independent Researcher, Birmingham, UK; Health Economics, Health and Population Sciences, University of Birmingham, Edgbaston, Birmingham, B15 2TT UK; School of Social and Community Medicine, University of Bristol, Canynge Hall, 39 Whatley Road, Bristol, BS8 2PS UK; Department of Nursing, Health and Population Sciences, University of Birmingham, Edgbaston, Birmingham, B15 2TT UK

**Keywords:** Organizational culture, Patients, People, Place, Theory, Realist evaluation

## Abstract

**Background:**

Organizational culture is considered by policy-makers, clinicians, health service managers and researchers to be a crucial mediator in the success of implementing health service redesign. It is a challenge to find a method to capture cultural issues that is both theoretically robust and meaningful to those working in the organizations concerned. As part of a comparative study of service redesign in three acute hospital organizations in England, UK, a framework for collecting data reflective of culture was developed that was informed by previous work in the field and social and cultural theory.

**Methods:**

As part of a larger mixed method comparative case study of hospital service redesign, informed by realist evaluation, the authors developed a framework for researching organisational culture during health service redesign and change. This article documents the development of the model, which involved an iterative process of data analysis, critical interdisciplinary discussion in the research team, and feedback from staff in the partner organisations. Data from semi-structured interviews with 77 key informants are used to illustrate the model.

**Results:**

In workshops with NHS partners to share and debate the early findings of the study, organizational culture was identified as a key concept to explore because it was perceived to underpin the whole redesign process. The Patients-People-Place framework for studying culture focuses on three thematic areas (‘domains’) and three levels of culture in which the data could be organised. The framework can be used to help explain the relationship between observable behaviours and cultural artefacts, the values and habits of social actors and the basic assumptions underpinning an organization’s culture in each domain.

**Conclusions:**

This paper makes a methodological contribution to the study of culture in health care organizations. It offers guidance and a practical approach to investigating the inherently complex phenomenon of culture in hospital organizations. The Patients-People-Place framework could be applied in other settings as a means of ensuring the three domains and three levels that are important to an organization’s culture are addressed in future health service research.

## Introduction

Organizational culture is broadly defined as ‘that which is shared by individuals within the organization—their beliefs, values, attitudes and norms of behaviour’ [1] and there is general agreement that it has a role in promoting some behaviours and blocking others [2,3]. However, as a concept, it is characterised by a lack of clarity arising primarily from the tension between whether culture is seen as something that an organization is (an emergent property of an organization that can be richly described in the interpretivist tradition), or something it has (a set of organizational attributes that can be measured in the positivist tradition) [1,4]. It is a challenge to find a method to capture cultural issues that is both theoretically robust and meaningful to those working in the organizations concerned. In this article, the interrelationship between organizational culture and approaches to service redesign is explored, drawing on comparative research in three English National Health Service (NHS) acute hospital Trusts. Culture emerged, in multiple and sometimes contradictory ways, in the early part of our research as one of the most important themes for further study. Thus, it became essential to develop a practical approach to the study of culture that could be used as the research moved into the next phase. The purpose of this article is to provide an account of how a framework was developed (the Patients-People-Place framework)—for the investigation of the interrelationship of culture and service redesign in the English NHS—to render the findings useful for researchers, NHS managers and clinicians. In order to do this, the paper is divided into five sections. First, the empirical and theoretical literature is summarised to set the project in context. Then the study is described, followed by an explanation of how the framework was developed. This is followed by examples of how the framework was applied to the data, and then the key points are re-revisited in the discussion. Finally, conclusions concerning the utility of the framework in accessing data that reveal the elements of organizational culture in health services research are presented.

## Background and related literature

### Service redesign

Government-led service redesign has become a constant feature of life in the English National Health Service (NHS) [5-7]. The rationale for service redesign is that reviewing current practices while identifying and removing obstacles will improve quality, efficiency and patient care [8]. Key reforms have focussed on the structural changes casting NHS Trusts as providers of care and strengthening the role of the organisations which commission services [9]; the establishment of ‘Foundation Trusts’ which have greater independence and freedom to manage their ‘business’ as determined by the board [10]; the use of Private Finance Initiatives (also known as Public Private Partnerships) designed to enable public hospitals to access private finance to fund capital projects [11], and encouraging Trusts to become ‘learning organisations’ to facilitate change processes to improve quality and safety [2,12]. Drawing on the work of the Institute for Healthcare Improvement, The NHS Plan [13], a major government policy document setting out a ten year plan of health reform, cited ‘redesign’ 17 times and raised expectations of a ‘system-wide programme of reform’ [14]. Many redesign methods developed in the manufacturing and private sectors have been applied to the health service, including Total Quality Management/Continuous Quality Improvement, Business process reengineering, Rapid cycle change, Lean thinking, and Six Sigma [15], and a key challenge to their successful application has been the failure to engage doctors and other clinicians in the change process [16] because, for instance, they feel that the ‘blue skies thinking’ produces goals that are out of reach and impractical [15,17]. In other cases, where successful ‘bottom-up’ change has taken place, it is generally locally based and ‘good’ practice fails to spread [14]. Overall, the evidence of the impact of the type of redesign programme promoted in the NHS is equivocal [18], and in this context redesign activity coexists with other policy reforms intended to incentivise behaviour to improve efficiency and quality, such as Payment by Results [19], the achievement of waiting list targets [20] and the imperative to increase patient safety [21]. However any major change or indeed service redesign involves a shift in the culture of the organization [22].

### Organizational culture

For almost a generation, the term culture has become commonplace in a wide range of literature concerned with health care organizations, and used to identify a range of issues. For example, the direction of a particular change has been articulated as the need to develop a ‘proactive health and safety culture’ [23], a ‘positive culture for public involvement’ [24], and a ‘culture of consensus’ [25]. It is often referred to in the context of change and research interest has centred on its role in this [26-28] along with service quality [29] and patient safety [30]. Most recently, the importance of culture was exemplified in the Francis Report [31] into the failings of the Mid-Staffordshire Hospital Trust in England. The need for cultural change culture runs through its 290 recommendations which advocating the need for a new NHS culture founded on care, openness, transparency and candour. Culture is often used to describe the general climate or feeling in an organization however there is no consensus on the meaning of the term [22,32-34]. This presents a challenge when attempting to operationalize the concept and research teams tend towards selecting a convenient definition according to their needs and sensitivities [35]. Mannion et al. [36] found seventy instruments and approaches in use for assessing organizational culture and that about a third of NHS organizations were using such an instrument as part of their clinical governance activity. The advantages and constraints of various cultures have been reported. For example, the benefits of participative cultures have been lauded [27] and the dangers of performance management cultures described [37]. There is general agreement that ‘culture has become something of a fixation for management’ [38] and it is necessary to refine the way(s) it is studied (at operational as well as strategic levels [37]). New and more sophisticated culture change models and frameworks are needed which allow for a multiplicity of competing cultures within an organization rather than assuming a monolithic culture which is sustained until overthrown by the new order [39]. Finally, it has been recognized for some time that there cannot be a culture unless there is a group that ‘owns’ it. Culture is embedded in groups and to understand organizational culture it is important to access those groups [40]. In the study reported here, the way different groups experienced and expressed culture was examined in order to learn more about the process of service redesign. However, in order to do this it was necessary to develop a way of accessing data on culture. The framework we developed for this purpose was informed by a consideration of the theoretical foundations on which we were basing our understanding of culture.

### Theoretical foundation

We intended to examine where culture was reciprocally related to those agents who enact it, also characterised as a ‘recursive’ approach [41]. Our starting point was the ‘levels of culture’ framework because it distinguishes between what is observable and what is not [40,42]. In addition there was the potential to operationalize these levels in the collection and analysis of data. On the surface level are the ‘artefacts and creations’ of culture, such as observable technology or behaviour patterns. Investigation at this level may reveal what is going on but not why. Such manifestations of culture reflect the ‘values’ which guide behaviour (second level), in terms of what is acceptable or not, which may be articulated by social actors, in research interviews for example. We include two important caveats here though: first, these values are neither static nor universal, and second, not all action is conscious, rational or reflexively informed; it is also influenced by habit. Habits are instinctive acts shaped by wider social structures that they reflect. We understand social structures as the institutions or societal norms that constrain the behaviour/praxis of individual social actors. The final level of culture, ‘basic assumptions,’ underpins these values and is founded on ontological and epistemological beliefs about the nature of reality, human relationships, and the relationship of humans with their environment. These are often pre-reflexive assumptions about the world, or what Bourdieu terms ‘doxic’ beliefs [43], that is ‘unquestioned beliefs, embodied in action and feeling but seldom formulated in words’ [44]. Consequently, this level of culture is difficult to access. It is often only when a crisis or conflict occurs, and people adopt a critical perspective to their world, that these basic assumptions are revealed on the surface and can be examined. Social actors and social structures, therefore, sit within a broader set of assumptions about the way the world works. This view of culture, drawing on the foundational work of Schein [42], offered the prospect of informing a practical and rigorous framework for accessing it in the study.

### The study

The study was funded by the National Institute for Healthcare Research (NIHR) as part of a ‘Collaborations for Leadership in Applied Health Research and Care’ (CLAHRC) infrastructure grant. This initiative sought to address the gap between research evidence being developed and its implementation in the NHS. Nine CLAHRCs were established in England, each in receipt of up to £10 m over five years (2008 – 2013) and were organised geographically. The framework reported here was developed as part of a project investigating health service redesign and its impact on three NHS Trusts in a region in England. Fundamental to the CLAHRC ethos is close working with NHS partner organizations to help ensure that the research informs future service redesign. The study was divided into two phases: the baseline was a comparative study of the ‘approach’ to service redesign in the three Trusts and the longitudinal phase, where service redesign activities in four specific clinical areas (‘tracers’) were evaluated [45]. We developed the patient-people-place framework for the study of organizational culture in the early stages of the study through the processes of data analysis, discussion of findings, recourse to the relevant literature (summarised earlier), feedback from Trust partners, and planning for the longitudinal phase.

The study was informed by principles of realist evaluation. The complex nature of health systems means that evaluating health service redesign is inherently challenging [15,46]. It is often not possible to isolate the effect of any individual change on outcomes, because it is part of an open system [47] and affected by a number of factors including culture. Outcome-based evaluation methodologies, such as randomised controlled trials, are unsuitable for studying complex causality and the influence of context on the success of such changes [48]. Formative process evaluations can address complexity using mixed methods and inform the development of further interventions [49], yet may lack rigour and fail to provide definitive conclusions about what ‘works’ [48]. Realist evaluation combines the best features of these contrasting approaches [50,51]. It concentrates on the context-mechanism-outcome pattern configuration to reveal what works for whom and under what circumstances [51]. Mechanisms (the cogs or working parts of an intervention) produce the effects. Context refers to the relevant features of the environment or conditions in which the mechanisms work. Outcome patterns are the intended (and unintended) consequences of a redesign programme; they are the product of different mechanisms in different contexts [51]. By focusing on the underlying elements of change (context and mechanisms), findings tend to be more generalizable, thereby increasing learning across policy, practice, and organizational boundaries [51]. In addition, the concern with context facilitates a dynamic and responsive approach to changes that may occur during the study. It was this element of the overall design that enabled us to recognise the need to develop a more robust way of accessing and organising data relating to the culture, or context, of the organisations we were investigating.

There have been criticisms and limitations reported in the literature of the realist approach [50,52,53], which have helped develop its use in evaluation practice over time. Although we were not directly evaluating specific interventions, a general realist approach informed the methodology adopted in the study. This was consistent with the intention to investigate service redesign prospectively and comparatively, within a changing context and in partnership with NHS colleagues [54]. The involvement of NHS clinical and managerial staff raised consciousness of research and embedded evaluation as a fundamental and inseparable part of the redesign process [55]. Within the realist approach, stakeholders are viewed as ‘fallible experts’ integral to the redesign programme and its evaluation, not merely as self-interested or unimportant [51]. A number of key stakeholders, namely our clinical partners, endorsed this need to marshal the data concerning organisational culture in a more systematic way.

### Ethical approval

The study was designated as Service Evaluation by the NHS National Research Ethics Service so not subject to approval by them. Ethical review was conducted by the University of Birmingham Ethical Review Committee (ERN_10-0034) and appropriate Research and Development Approval was secured at partner Trusts.

### Setting

The three (pseudonymized) hospital Trusts were selected as case studies from a region of England, UK. It is likely that these organisations may be recognisable to those familiar with the region or the CLAHRC; however these generic descriptive pseudonyms do afford a degree of anonymity in the wider dissemination of the findings. They met three criteria: geographical proximity to each other; all were undergoing significant spatial-structural changes and redesign of services, such as building new hospital facilities or moving services into the community; and were willing to be involved in the study. University Trust is a large teaching hospital. Only about half (54%) of its admissions were from its host (Primary Care Trust) PCT, because it is a tertiary centre attracting admissions from all over England. It was formed from the merger of two hospitals fifteen years ago and was on two sites. It was relocating to a new, single, purpose-built hospital during the research. Urban Trust is a large organization in an urban setting, the result of a merger of two district hospitals seven years ago and offers some specialist tertiary services. However, the majority of its admissions (79%) are from its two local PCTs. Town Trust is a district general hospital, with fewer tertiary services, and most (86%) of its admissions are from its host PCT. Part way through the study, the PCTs were dissolved and replaced with clinical commissioning groups, due to changes in national government policy.

### Data collection

A mixed method, case study methodology was adopted. During the baseline phase, semi-structured interviews with 77 key informants were conducted. In addition, although these data are not reported in this article, hospital level data were compared (such as Care Quality Commission ratings, published staff survey data and analysis of length of stay) and participant-observation of meetings related to service redesign activity was undertaken. The research team attended fortnightly meetings to debrief and reflect on post-interview and observation notes and discuss emerging findings. For the interviews, a combination of purposive and snowball sampling [56] was used with the aim of obtaining a strategic view of the respective organizations by interviewing senior executives, managers and clinicians with responsibility for specific parts of the organization, including representatives from the medical, nursing and allied health professions, trades unions, patient representatives, and executive representatives from associated local acute Trusts. An interview topic guide and prompts were used to structure the discussion, drawing on information from existing literature and the research questions. The interviewers encouraged respondents to discuss their perceptions and experiences freely by assuring that no data extracts would be directly attributable to any individual.

### Data analysis

The interview transcripts were analysed using an inductive Framework Method approach [57,58]. All interviews were digitally audio-recorded and transcribed verbatim. Four interviewers independently read and open coded six transcripts. Discussion of these led to the development of a working analytical framework grouped into the following themes (Trust objectives, national context, regional/local context, internal structures, and processes). Interpretation of the data was conducted in team meetings, which often involved vigorous debate where the different disciplinary perspectives the team members brought to the process were explored and, if necessary, challenged. The range of backgrounds—clinical practice (medical, surgical and nursing), sociology, health service management, health economics, and psychology—provided a rich set of disciplinary lenses through which to interpret the data. This process, along with the regular meetings with staff from the partner organisations consistently identified that respondents made frequent references to culture and its importance. This observation, along with the prominence of culture as a concept in the policy and research literature prompted the team to review its approach to investigating this element of service redesign. Consequently during the course of the baseline phase of the study, the team developed a framework for studying culture that was rooted in the empirical data being collected and analysed, informed by previous work in the field (see related literature and theoretical foundations sections), including social and cultural theory, and responsive to the concerns and priorities of our NHS partners. This framework guided the methods and data collection instruments used to explore culture in the longitudinal phase. Using the main framework approach as a guide [54,55], a matrix was developed to identify the categories of data that relate to the three levels/domains of culture [57,58]. This was then populated with data extracts to facilitate the exploration of the impact of culture on service redesign in the trusts.

### Domains of culture: the patients-people-place framework

Three ‘domains’ where organizational culture is played out and enacted were identified to ensure the data collection was consistent. The framework was informed by re-analysis of the data from the baseline phase to test its utility, and the suggestions made by staff about areas to investigate further following feedback of the baseline results. The domains are patients, people, and place, which are summarized below with illustrative examples from the data identified through application of the framework. Table 1 summarizes the methods and types of data that can be used to investigate each level of culture within each of the three domains.

**Table 1:** **Indicative types of data (** ***and methods for collecting data)*** **for each level and domain of culture in a hospital change programme**

**Level/Domain of culture**	**Patients**	**People**	**Place**
Observable behaviour and artefacts *(audits, surveys, patient satisfaction monitoring, observation of activities undertaken as part of a redesign programme, content analysis of information and communication)*	Patient information leaflets/posters; lay members of boards; methods for consultation/involvement in redesign initiatives	Frequency and extent of consultation with or full involvement of stakeholders in decision making; modes and content of communication about potential and actual changes to the service; management structures	New building layout and facilities; reallocation of services between primary, secondary, tertiary or community health spaces; development of day surgery units; tension between consolidation and decentralization of services
Values and habits of social actors *(phenomenological interviewing, participant-observation)*	Value statements from staff and patients about initiatives to involve patients; ways of talking about everyday practice and change	Value statements from senior and frontline staff; deployment of ‘change agents’ to show where perceived barriers to change are; views on role of government policy	Associations made between buildings and quality; views on community; co-design practices for new clinical space
Basic assumptions *(theory, discourse analysis, ethnography)*	Power relations between staff and patients; professional and organisational structures; ideologies of care	Power relationships between clinicians and management; organisational hierarchies; professional divisions of labour	Ideologies of progress, technological development and modernization; communities of practice

### Patients

The focus of this domain is the ‘perceived importance and practical role of patients in service redesign’ [59]. This includes the attitudes to and application of the concept of patient-centred care in practice, the involvement/engagement of patients in service redesign, and the methods for assessing the quality of patient care. While an emphasis on quality of care and the promotion of efficient services was central to the strategic vision of all three hospitals, the way these aims were articulated varied considerably and revealed differences in assumptions and values in relation to care priorities and the patient role. In all three Trusts, the concept of ‘patient-centred care’ or ‘improving patient experience’ was identified as an aim or principle underlying service redesign, with some interviewees going into more detail (‘building the service around the patient’; ‘patient journeys can be unnecessarily long and costly’). However, although patient-centredness’ was espoused as a value—‘[we have to] focus on the fact that there is a patient as the end of every sample [we process]’; ‘everything we do… it has got to improve the quality of care for patients, otherwise we’re not doing it’—there was little elaboration on what this meant in practice and lack of understanding of key concepts such as ‘engagement’ and ‘consultation’ was evident. At University Trust, for example, despite some clear signs of active patient engagement (such as the establishment of a ‘Patients’ Council’), when the concept of patient-centred care was mentioned by interviewees it was generally used to refer to the patient as the ‘object’ of care rather than a ‘subject’ with agency and views [59]. In other words, there was no discussion of passing control of their ‘journey’ to patients and more about planning the efficient running of the organization around them. In most cases, when staff talked about patients it was in terms that characterised them as passive, abstract and disembodied.

In much of the literature and policy guidance, ‘quality’ is defined in terms of quantitative outcome measures rather than qualitative assessments [60]. Yet many interviewees, particularly those from nursing backgrounds, acknowledged that good patient experiences were not solely related to clinical outcomes. One senior nurse explained that, ‘using outcomes that are important to patients help determine how successful we are’, while another said ‘metrics are not enough’. At University Trust there was a specific IT initiative to collect real-time patient feedback, because the National Patient Survey was seen as taking too long to deliver results.

Many respondents from clinical and managerial backgrounds believed that the local population did not really understand how the health system worked, citing inappropriate use of emergency services as an example of the effect this had [59]. One interviewee from Town Trust went further and expressed concern that the public and patients did not have sufficient expertise to be involved in service redesign: ‘sometimes it is just better to present it to them [patients] and ask them how they feel at the end.’ By contrast, in the current policy climate that emphasizes patient choice and competition, ‘marketing’ the service to the public (in order to pre-empt criticism in the press for example), and communicating improvements and changes to service-users were recognised as important. For example changing the configuration of the two main hospital sites into ‘hot’ (taking emergency admissions) and ‘cold’ (planned cases) at Urban Trust, was problematic because patients arrived at the wrong place for appointments: ‘the natural tendency is for patients to vote with their feet and come to a local A&E [emergency department].’ This highlights how the espoused commitment to ‘patient centredness’ was difficult to achieve in practice [59] and reflects how the deeper levels of culture can be accessed by using the framework to ensure these issues are explored.

### People

This domain encompasses how different social actors interact to produce service redesign. In our data, the focus was on the various professions—medicine, nursing, and the allied health professions, as well as managers, administrators, porters, and domestic staff who worked in the organizations. However, there were also instances where other actors were referred to including the public, service users, patients, policy makers, journalists, and academics. A central theme that emerged from this domain in our data was the extent to which change was driven by people at the top, or emerged ‘bottom-up’ through the organization. In the three Trusts we studied there were clear differences in approach. At Urban Trust, the engagement of staff in decision-making was a core principle, underpinned by an award winning staff engagement programme. It worked, according to one clinician, because ‘the aim of the project had been made clear and the principle is sound.’ When staff members proposed solutions to organizational problems that were then implemented they felt valued. It was suggested that happy staff led to happy patients, a principle endorsed in the Boorman report on staff wellbeing in the NHS [61].

At Town Trust, by contrast, the Executive team was experiencing difficulties engaging staff. The clinicians explained it was being done mainly through the introduction of the clinical director role. With regard to the non-medical workforce, an Executive Director explained, ‘what we’re trying to do there is engage with them to show that we’re all part of the same organization, and to celebrate their successes, but also to get them so that they understand the strategic imperative of the organization.’ This was a top-down approach and there was no mechanism described for ensuring the ‘strategic imperative’ was informed by staff views. There were also problems with communication, distrust, and rumours reflecting the particular type of people-culture in the trust. ‘What they [the Executive Board] want to achieve and people down there think they want to achieve is very different.’ This division was exacerbated by, what one manager described as the ‘wasp-waisted’ management structure, with its stricture at middle management level, stifling two way communication.

At University Trust a more hybrid approach had been developed. As one executive team member explained ‘what does the best look like [for each service] … we just go through the process to build up [the] clinical strategy from the bottom up within [our Trust] framework … We then cross cut their plans with our values.’ Another member of the executive team explained: ‘there’s a lot of executive steer … but I also think there’s a lot of drive from the specialties, who know their services need to change, have real vision for how they want to do it, because they’ve been helped along with that … Somewhere in the middle there makes that sort of tension which works … [the] grit in the oyster.’ Clinical staff were given ownership of their change because they were enabled to identify their own targets, budgets were devolved and change champions identified and supported.

Data charting the response of the trusts to external policy drivers also demonstrated the differences in cultures. A number of senior managers at University Trust maintained that they were ‘absolutely never driven by national policy.’ However, the hospital invariably met national targets and so was capable of ‘playing the game.’ Moreover, they felt part of their role was ‘to shield the people in the organization from as much of the lunacy that the outside world and the NHS is putting upon us.’ Bourdieu observes that ‘nothing is simultaneously freer and more constrained than the action of the good player’ [43]. Although he is referring to individual agents, the observation can be applied to explain organizational behaviour. The ‘good’ NHS organization is able to mediate between national policy and the values and ambitions of individual clinicians and clinical teams, to produce strategic action that feels ‘top-down’ to those at the top and ‘bottom-up’ to those lower down the hierarchy. Despite this commitment to engaging staff, there was a sense within the Executive team that negative views would not be tolerated indefinitely: ‘if we’re to achieve real transformational change here then … we need to start saying to people that don’t sign up to our values and behaviours that they actually aren’t welcome here.’ In our study, a crucial dynamic in this domain was the relationship between clinicians and managers. Medical professionals are recognised as a powerful group in the health system and, and in the past have resisted the introduction of management approaches in the health service that constrain their activity [62,63]. Differences in priorities between the two groups were reported. An Executive team member from Town Trust stated: ‘Doctors need to understand they can’t do anything without money; managers need to understand there’s no point doing anything about money if we haven’t got the quality right.’ There was also evidence of scepticism on the part of clinical staff with regard to managers’ intentions in all of the Trusts. At Town Trust, one Executive Director said, ‘The other key change for me is to get the clinicians involved in caring for the hospital rather than just the patient in front of them.’ Conversely, one consultant at Urban Trust, where organic and engaged change was generally favoured, criticized management for its failure to stand up to clinicians: ‘you don’t have the kind of persuasiveness of management to make it seem anything other than something that’s imposed … I think there’s an awful lot of avoidance of conflict, now whether you can actually avoid the conflict and get the end results, I’m not sure.’ In all three Trusts, leadership was identified as crucial to innovation [64] and the development of a good ‘people culture,’ which is consistent with national policy [65-69].

The importance of leaders having clinical credibility, the development of leadership skills throughout the organization, and the need to address the ‘problem’ of middle management where change often stalled [69,70] were highlighted. The quality and style of the executive team and the Chief Executive, were also seen as a key factor in whether or not the culture of the organization was conducive to change. At the heart of the people-culture domain in the context of the model, is the attention it directs to who drives change, and how the power relations inside and outside the organization are played out during the change process. Ensuring the key relationships in the delivery of health care are explored, through application of the framework, uncovers this dimension of culture and its influence on service redesign.

### Place

The focus of the place-culture domain is the location of care, the meanings embedded in different health spaces, and the practices enacted there. Place affects clinical care services and the possibilities for service redesign. Accounts of the hospital redevelopment (new buildings in particular) as an icon of quality, effectiveness and progress were examined as an important theme in the interviews, as identified by application of the framework. However when examining the data it became clear that there was also a recognition that the hospital as a health space par excellence was being challenged by the drive for more local and patient-centred care. The development of new hospital facilities at University and Town Trusts was mentioned by most of the interviewees. The need to move services to the community to manage demand and reduce lengths of stay, particularly for elderly patients, was acknowledged. At Urban Trust, the ‘new hospital’ was an aspiration and the main focus of activity was on the relocation of services across the full range of primary, secondary, and community health spaces (known as the Collaborative Care Programme (CCP is a pseudonym)). Nonetheless, the promise of a new hospital at some point in the future was regarded as a strong incentive to engage clinicians in the CCP as a focus for service redesign. As a senior doctor put it: I’m just giving you my perception… that effectively the reason that we’re doing this [CCP] is to get an argument for a new hospital to be built … for the relevant amount of activity to be released for the community to afford that.’ More generally, although some were positive about this aspiration, other respondents were not always persuaded that moving services to the community would be clinically or financially beneficial. However, the relocation of services (whether to the community or to a new hospital) was seen as an ‘opportunity’ and ‘focus’ for service redesign. This was because change would be unavoidable with the opening of new facilities, such as a ‘47 hour’ unit at Town Trust, or because practice would need to change to deliver services in new wards at University Trust simply because they were larger and had a different staff configuration.

Idealised accounts of a service where there was improved ‘flow’ and more ‘efficient’ ‘sustainable’ patient pathways were given in many of the interviews. However respondents at Urban Trust felt the priority had been the relocation of services to the community rather than redesign, which a number criticised as ‘drag and drop,’ and felt it was a missed opportunity. Effective planning was seen by staff at all three Trusts as central to the success of service redesign alongside a physical move, including the implementation of as much of the redesign as feasible before the actual relocation took place. This illustrates how the links between the different components of the Patient-People-Place framework can be used to interpret culture. Consultation and engagement in the design of the new facilities was vital, and there were two benefits of this: first on practical level, staff could ensure that the fine details of the new wards would be suitable, such as ‘where sockets go’ or the ‘height of commodes,’ as one nurse from University Trust commented. Second, the organizational and professional identity, bound up with ‘place’ could be considered. Health spaces produce communities, and the potential disruption that might result from changing them was a cause for concern, as this senior nurse from University Trust explained:

‘what’s very interesting about hospitals is that I think they’re quite evocative places, people talk fondly about all the ones that have been knocked down …, which everybody will tell you, in the cold light of day, were a complete nightmare to work in … But that sense of community is the thing that leaves people exposed … the important thing for us is to let people celebrate their sense of community, and bring them into the centre of the next community, so that mixing of people [staff] early on, which we’ve begun to do, is to try to get teams to meet.’

At Urban Trust, the two-site structure was recognised as being ‘divisive’ and a barrier to integrated working. At Town Trust, the hospital as the geographical and metaphorical centre of the community was important (as a major employer and source of pride). Despite the acknowledgement that redesign and relocation were necessary, the new hospitals were seen as good things in themselves. The idea of a ‘shiny new’ building was frequently evoked, and it was argued that this would be of benefit to patients, who would have a better experience, and to staff, as a morale boost. At Town Trust, the new hospital was also seen as a guarantee that there would continue to be a hospital in the town, serving the local population. However enthusiasm for the new premises was equivocal, generating uncertainty and instability for some staff, particularly at Town Trust, who had concerns about the financial viability of the new facilities and who were not reassured that their jobs would be safe. A final dimension of the place domain concerns the politics of space; essentially, who gets what in terms of accommodation. Numerous examples of these political tensions are evident in the data. For example, the staff in a specialist hospital adjacent to University Trust who wanted a covered walkway to connect the new hospital to their building, to signal their partnership. The dissolving boundaries of space in the new layout of University Trust, where theatres were booked ‘like a hotel’ rather than having traditional sessions, brought out the ‘notoriously protective and territorial’ nature of surgeons, according to a senior manager. In short, in the place domain there were two simultaneous drivers in tension with one another, consolidation and decentralisation, and both were mediated by power relations. Using the framework to interrogate the data for material concerning ‘place’ helped ensure that key elements of culture were examined and demonstrated that it could be applied to data sets in the next phase of the study, and indeed other studies.

## Discussion

These three domains in which culture was enacted—patients, people and place—constitute a practical framework for identifying and linking more manifest applications of culture in organizations. Each domain can be explored at different levels of culture—artefacts, values and habits, and basic assumptions at work in the setting (see Table 1). The data presented above are illustrative (not comprehensive) of the sort of findings that can be located in each domain, albeit in more depth, through use of the framework. In the longitudinal phase of the study, 12 different services (4 types of service × 3 hospital Trusts), were evaluated prospectively during the process of service redesign. The focus on culture and how it is evident in the tracer services was maintained through recourse to the Patients-People-Place framework of organizational culture. The interview schedules were constructed to ensure attention was directed to these areas and the importance of context recognised. Our interpretation of culture will be filtered through our broader realist concerns with the context in which organizations function, the organization itself (i.e. its structures) and organizational processes and outcomes [71], including the interface between the different levels of the organization, as well as complex causality. With respect to the wider context, the financial climate and constraints on government spending [72] are affecting all of the three Trusts, but in different ways [41]. Each clinician, manager, patient and organization will be more influenced by some social structures or policies than others, and may take the opportunity to redesign services in different ways [41]. So while there may be a set of contextual factors that can be observed and recorded, the way those factors affect outcomes depends on the particular actor’s view, as well as the mechanisms used to bring change about. This is also particularly dependent on leaders who through their symbolic actions communicate what is important and shape change [30]. The organisation then sets the parameters for the kinds of local cultures that are possible, i.e. it constrains some possibilities and produces others. The organizational culture also intersects with other cultures, such as medical and nursing professional cultures, where there may be tensions between the values and habits of individuals in management and those in clinical work.

Consideration of different domains and levels of culture can point the way not only to explanation but the means of organizational change. Konteh et al. [4] found that patients and their representatives could be a force for cultural change if staff work in partnership with them. To the extent that groups such as the medical profession have the power to subvert or undermine service redesign, an organizational culture which seeks to either align redesign objectives with their values, or successfully confronts their cultural values, has been recognised as essential to successful change [14]. In our study, the efforts in one trust (Urban) to actively engage staff in the re-design process indicates the sort of action required to address constraints on change. However, in a similar engagement programme staff reacted in a variety of ways with some feeling more content and involved, others sought to leave the organization, and a number ignored the programme and pursued a course of ‘quiet resistance’ [73]. This emphasises the importance of context and reinforces the need to approach the study of culture in a systematic way in order to uncover such differences. The Patients-People-Place framework provides a practical means of doing this. Mannion et al. [39], called for new and more sophisticated culture change models and frameworks which allow for a multiplicity of competing cultures within an organization. The intention is that the framework, developed in response to a need to access data reflecting culture in a systematic way, can be used to build accounts of culture(s). In a sense this aim is fairly straightforward and rests on the assumption that the main characteristics of an organization’s culture can be described and assessed in terms of their functional contribution to broader managerial and organizational objectives [28]. However it remains an elusive concept, fraught with competing interpretations and lacking a consensual definition [22,29]. In view of this application of the Patients-People-Place framework is offered as a practical way of addressing this challenge, which has been useful in our study to date and may be of value to others involved in similar work. For our partners, the key pragmatic question was ‘What elements of culture cannot be ignored during service redesign?’ and through application of the framework we were able to share the examples of this noted earlier. However it must be acknowledged that even with the conduct of rigorous studies we can only make statements about elements of culture, not its entirety [42].

## Conclusion

This framework for studying culture in health care organizations comparatively during change was developed through the integration of existing theory, review of empirical evidence from the early stages of our study, and involvement from our health service collaborators. It incorporates three domains—patients, people, and place—and is rooted in a recursive and realist approach to studying social life, with an explicit sensitivity to context. It is also consistent with the ‘theory testing’ element of Pawson’s work [74], which in turn is reinforced by the underlying realist approach pursued in the study as a whole. Concepts for understanding culture in organizations have value only when they derive from observation of real behaviour in organizations, when they make sense of organizational data, and when they are definite enough to generate further study [42]. The approach taken to operationalizing the amorphous and complex nature of culture in this particular setting was an attempt to meet these requirements. The intention is that it presents a helpful framework for grounding the concept in its context, whilst highlighting how specific aspects can be accessed, observed and reported. As with any emerging theory, the Patients-People-Place framework for studying organizational culture requires further refinement and testing. This now assumes even greater importance because as Davies and Mannion [75] argue more sophisticated understandings of culture and an appreciation of the policy in shaping these is needed if healthcare failings are to be tackled.
